# Remote assessment via video evaluation (RAVVE): a pilot study to trial video-enabled peer feedback on clinical performance

**DOI:** 10.1186/s12909-019-1905-3

**Published:** 2019-12-18

**Authors:** Kendall Ho, Christopher Yao, Helen Novak Lauscher, Barry E. Koehler, Kamran Shojania, Shahin Jamal, David Collins, Raheem Kherani, Graydon Meneilly, Kevin Eva

**Affiliations:** 1 Digital Emergency Medicine, Department of Emergency Medicine, Faculty of Medicine, University of British Columbia, 818 West 10th Avenue (3rd Floor), Vancouver, BC V5Z 1M9 Canada; 2 Division of Rheumatology, Department of Medicine, Faculty of Medicine, University of British Columbia, Gordon and Leslie Diamond Health Care Centre, 10th Floor – 2775 Laurel Street, Vancouver, BC V5Z 1M9 Canada; 3 Division of Geriatric Medicine, Department of Medicine, Faculty of Medicine, University of British Columbia, 10th Floor – 2775 Laurel Street, Vancouver, BC V5Z 1M9 Canada; 4 Centre for Health Education Scholarship, Department of Medicine, Faculty of Medicine, University of British Columbia, P.A. Woodward Instructional Resources Centre, 429 – 2194 Health Sciences Mall, Vancouver, BC V6T 1Z3 Canada

**Keywords:** Physician, Peer feedback, Self-evaluation, Reflective practice, Video feedback, Medical education

## Abstract

**Background:**

Video review processes for evaluation and coaching are often incorporated into medical education as a means to accurately capture physician-patient interactions. Compared to direct observation they offer the advantage of overcoming many logistical challenges. However, the suitability and viability of using video-based peer consultations for professional development requires further investigation. This study aims to explore the acceptability and feasibility of video-based peer feedback to support professional development and quality improvement in patient care.

**Methods:**

Five rheumatologists each provided four videos of patient consultations. Peers evaluated the videos using five-point scales, providing annotations in the video recordings, and offering recommendations. The rheumatologists reviewed the videos of their own four patient interactions along with the feedback. They were asked to document if they would make practice changes based on the feedback. Focus groups were conducted and analysed to explore the effectiveness of video-based peer feedback in assisting physicians to improve clinical practice.

**Results:**

Participants felt the video-based feedback provided accurate and detailed information in a more convenient, less intrusive manner than direct observation. Observations made through video review enabled participants to evaluate more detailed information than a chart review alone. Participants believed that reviewing recorded consultations allowed them to reflect on their practice and gain insight into alternative communication methods.

**Conclusions:**

Video-based peer feedback and self-review of clinical performance is an acceptable and pragmatic approach to support professional development and improve clinical care among peer clinicians. Further investigation into the effectiveness of this approach is needed.

## Background

Physician practice evaluation to support maintenance of competence is a complex process that can include multiple components, from charting to patient encounters. Direct observation of clinical encounters between physicians and their patients can be a valuable technique to evaluate clinical performance and communication skills. It may also enhance physicians’ self-reflection and contribute to professional development and quality improvement.

Implementing direct observation techniques, however, can be challenging. Direct observation requires the simultaneous availability and participation of an evaluator, the physician, and willing patients. Also, the presence of an outside observer can unintentionally alter the dynamic of physician-patient interactions, as both physicians and patients can be conscious of the presence of an outside observer. Consequently, physicians or patients might act artificially, preventing the observer from examining a more natural interchange between the physician and his/her patients. Direct observation is also labour-intensive for the examiner, requiring travel to different locations to carry out the assessment. Such logistical issues often make direct observation infeasible, particularly for physicians practicing in non-urban locations.

Video-based feedback can conceivably contribute to continuing professional development by retaining the benefits of direct observation while simultaneously decreasing many of the above-mentioned challenges. Video-based feedback – whereby the encounter of a learner with a patient is recorded and sent to a reviewer for comments and evaluation – has been applied extensively to medical and non-medical education with the objective of improving individual practical skills like communication or surgical techniques [1–7]. Video-based evaluations have the potential to be advantageous in the following ways: a) reviewers do not intrude physically during physician-patient interactions, making the encounters more natural and authentic, thereby reducing observer effects; b) the physician being assessed can interpret the observer’s critique while watching his/her own performance on the recorded video rather than having to rely upon memory; c) reviewers need not be available at the same time and physical location as the physician being evaluated, allowing the assessment to take place asynchronously; d) the record of the assessment and the annotations can easily be kept for future or additional reviews if needed; and, e) the format allows time for deliberate self-evaluation and reflection [8].

However, video-based peer feedback also has potential challenges: a) physicians may not engage with the feedback as readily in a video format that is deprived of interactive dialogues with the reviewers; b) the person being observed is not able to probe deeply into the reviewers’ critique in a manner that could help them better understand the issues and improve upon them; c) asynchronous assessment delays feedback, thereby reducing its timeliness and potentially its effectiveness; and, d) video recording might still be seen as intrusive because patients or physicians might not like to be recorded [8].

Video-based feedback has been researched previously at our institution to evaluate its effectiveness in teaching medical students. In this context, studies found that video annotated feedback helped students identify specific areas of strength and weakness and increased the overall acceptance of scores [6, 9]. Additional research has demonstrated positive outcomes in the use of video-based assessments of medical students by physician mentors, including enrichment of students’ learning from clinical settings, exposure of students to a larger number of mentors, and improving the ability of students to recall their own performances [10].

For practicing clinicians, continuing professional development is vital to ensure quality and up-to-date practice in patient care. The capacity for video-based review to support the evaluation of clinical performance and provide feedback, to yield opportunities for self-reflection on clinical approaches, and to allow consideration of alternative approaches to clinical care, holds great potential for improving continuing professional development. However, there is limited understanding of whether video-based feedback would be used by physicians in practice as a method for continuing professional development and quality improvement [8].

To investigate the utility of video-based feedback among peer clinicians as a feasible and acceptable method to support performance improvement, we conducted a qualitative study, named Remote Assessment via Video Evaluation (RAVVE), using a participatory action research model. The overall objective was to explore hypothesized benefits and drawbacks of video-based consultations and whether a group of clinician peers would find this a helpful approach for their continuing professional development and quality improvement.

## Methods

### Procedures

This study used a mixed methods approach, based on the principles of participatory action research [11]. Such research uses naturalistic inquiry to engage participants, is grounded in firsthand experience, and is action-oriented. Therefore, we enrolled practicing physicians as first-person participants and peer reviewers during the design, conduct, and reporting of the research. Rheumatology was selected as the discipline from which to recruit because it is a domain in which patients present with both chronic and acute problems to an office setting in which video cameras could be set up unobtrusively. As such, five practising rheumatologists were engaged with the researchers to develop, implement, and evaluate all phases of this project. We purposively sampled physicians with experience in education and assessment to allow for a fulsome critique and evaluation of the video based system. They represented diversity in years of practice, gender, and practice profile. They were similar, however, in that all were active rheumatologists working in an urban setting and all were involved in medical education for peers, medical residents and students. These features ensured that each participant brought a depth of contextual knowledge to the study that could strengthen the credibility of the findings among their colleagues, a strategy that is associated with greater acceptance and long-term adoption among stakeholders [12–14]. In keeping with the participatory action method, a critical constructivist approach was used, engaging participants with the techniques and activities afforded by the video review system and enabling them to reflect on their own experience with it. Throughout the procedure the clinician co-investigators were asked to approach the activity with a critical lens, sharing and documenting their observations as a cohesive group of colleagues.

Ethics approval was obtained from the university’s Behavioural Research Ethics Board prior to the study. As per ethics requirements, all participating physicians provided signed consent to participate.

#### Phase 1: protocol development

The clinicians and researchers participated in a focus group to contemplate contextual constraints and generate consensus on how a video-based peer feedback process could be conducted inconspicuously and efficiently while also deciding what tools would be most useful to evaluate each recording.

#### Phase 2a: proof of concept testing – participant recruitment

Each physician recruited a convenience sample of four patients to participate. On the day of a medical appointment, the physician office assistant or a co-investigator presented, explained, and reviewed the study protocol with the patient. The patient provided written informed consent to allow video recording of their clinical consultation and the sharing of the patient’s chart among co-investigators. Although selected through convenience sampling, enough patients were recruited to provide the variety and content required to adequately allow for critique of the video assessment method.

#### Phase 2b: proof of concept testing – peer evaluation and feedback

All five rheumatologists recorded and provided four videos of patient consultations, ranging from 10 to 20 min, along with consultation notes for the recorded visit and prior visits relevant to the current visit. This number allowed for sufficient diversity of observation while also being deemed feasible for the physician co-investigators.

To conduct the video review, each reviewing physician received access to two videos of patients from each of the other four physicians. As the initial step, for each peer, the reviewing physician reviewed one video along with the provided consultation notes, completed a questionnaire (described below) to evaluate the performance observed, annotated the video using a software program located on a secure server, and provided a narrative outlining exemplary practices and recommendations for change. Two weeks later, this process was repeated for the second patient video received from each peer physician.

**Fig. 1:**
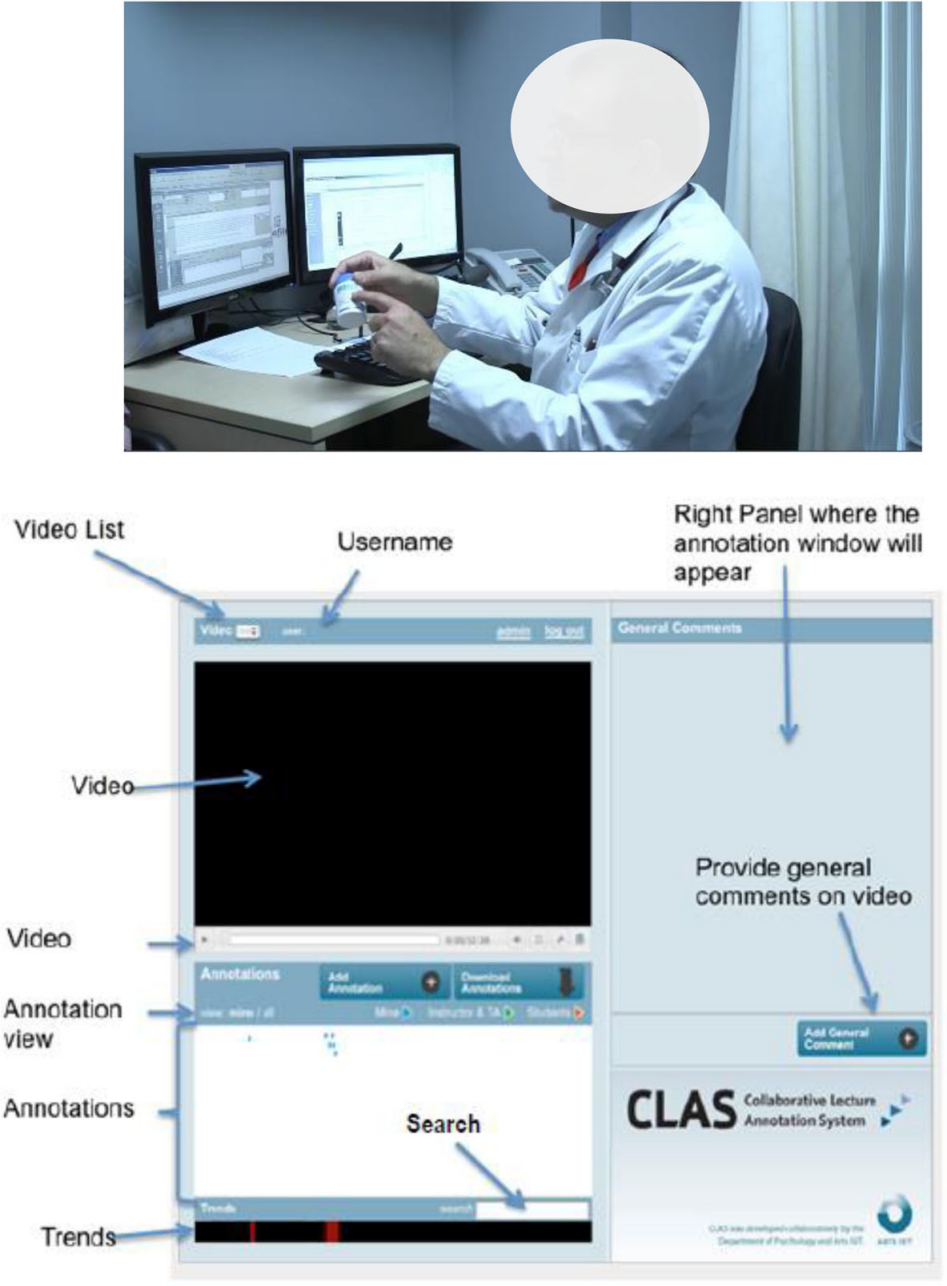
Video recording and software interface in the feedback and review processes

All videos were shared with physicians and researchers using a password protected and private web platform that enabled the research team to control access and deliver specific materials only to those who needed access. The video platform and software allowed feedback from the reviewers to be embedded directly into the video at corresponding time stamps (see Fig. 1 for the layout of the software interface).

#### Phase 2c: proof of concept testing – reflection

Two weeks after all videos were reviewed and annotated, each physician was asked to review the videos of their own four patients together with the associated feedback. To avoid bias, all physicians were required to provide their feedback and evaluation of others’ videos before they obtained feedback on their own cases. Time delays between each phase allowed for processing, preparation and distribution of videos, and sufficient time for participants to engage in the study amidst their busy clinical and teaching schedules.

#### Phase 3: post-pilot focus group

To complete the study, a focus group was conducted to gather information about the project and the protocol design, to obtain participants’ feedback on the utility of this exercise in helping them with their clinical practices, and to record their recommendations for how this approach could be used in the future for peer assessment. A semi-structured set of questions was developed to facilitate the discussion. Content analysis was conducted using a constant comparative approach. The focus group recording was transcribed and coded by a member of the research team to enable generation of themes and sub-themes.

To ensure the credibility of the findings, each participant reviewed and had an opportunity to edit the focus group summary. They were informed that collaborative discussion and co-editing of the summary was part of the analytic process. Conflicting edits or disagreements in interpretation were discussed, but all perspectives were included as part of the analysis. During review, participants were also given the opportunity to enrich the interpretation of the perspectives they expressed. Engaging in such verification maximized the richness and depth of our understanding of participants’ experience.

### Data collection tools

#### Medical colleague questionnaire

The feedback questionnaire that was used to evaluate each patient encounter was developed through the physician consultation process. To begin, the Medical Colleague Questionnaire used during Multisource Feedback by the College of Physicians and Surgeons of Alberta [15] was shared with the group and discussed. The questionnaire includes 31 performance items rated using a 5 point Likert type scale from “among the worst” to “among the best”. An “unable to assess” option is also available for each item. The questionnaire includes subscales focused on several areas of practice: consultation and communication, patient interaction, clinical competence, professionalism, and psychosocial patient management. Initial questions on the survey required the reviewer to describe their professional relationship with the person being reviewed (i.e., peer, consultant, referring physician) and to indicate how well they know the individual being assessed (i.e., not at all, not well, somewhat, well, very well).

#### Participant perceptions of feedback received

An 18-item questionnaire was also developed to enable participants to rate the feedback provided by their peers (see Table 2). All questions were presented with a five-point scale ranging from 1 (strongly disagree) to 5 (strongly agree).

## Results

This study took place from January 2013 to March 2014. It received ethics approval (certificate number H13–00590) from the university’s behavioural ethics review board prior to its conduct.

### Participants

Five rheumatologists (4 males, 1 female) and 20 patients participated in the study. All but one of the 20 video recordings were reviewed twice by peer physicians. When asked to describe the type of relationship maintained with the physician being reviewed, the physicians most often identified themselves as a “peer”. In only two cases did the physician regard the individual being reviewed as a “consultant”. As previously stated, all five were actively involved in education and known to have an interest in the area of technology enabled pedagogy, thereby allowing them to act as key informants in a participatory action research process.

### Phase 1: patient consultation video recording protocol

For the patient consultation recordings, the clinicians decided to engage patients that were returning for clinical follow-up rather than new patients cases because: a) physician-patient rapport had already been established, b) such patients would likely be less anxious, c) more of these patients could be seen in less time, and d) the physical exposure of patients during examination was likely to be more limited at follow-up.

As described in the Methods, a commonly used multi-source feedback questionnaire was presented to stimulate discussion regarding what could potentially be evaluated with video-based review and consensus-building was used to generate a modified questionnaire. After discussion of the context and likely patient scenarios it was concluded that 17 items (see Table 1) from the 31-item questionnaire would be retained and that open-ended questions regarding positive and negative aspects of performance would be added. The rating scales from the original questionnaire were kept.

**Table 1:** Physician Ratings

	N Valid	N Missing	N “Unable to Assess”	Mean	Std. Error of Mean	Median	S.D.	Minimum	Maximum
1. Communicates effectively with patients	59	0	0	4.56	0.07	5	0.57	3	5
2. Communicates effectively with patients’ families*	59	0	36	4.35	0.12	4	0.57	3	5
3. Within range of services provided by this physician, he/she demonstrates appropriate judgement	59	0	1	4.69	0.07	5	0.50	3	5
4. Selects diagnostic tests appropriately	59	0	9	4.46	0.09	4.5	0.61	2	5
5. Critically assesses diagnostic information	58	1	3	4.58	0.08	5	0.57	3	5
6. Assesses and evaluates potential toxicity of therapeutics	58	1	7	4.55	0.09	5	0.61	3	5
7. Provides appropriate monitoring of therapeutics	58	1	5	4.58	0.08	5	0.57	3	5
8. Assesses burden of inflammatory diseases	59	0	5	4.50	0.07	5	0.54	3	5
9. Selects the appropriate treatment	59	0	0	4.59	0.07	5	0.56	3	5
10. Maintains quality medical records	58	1	3	4.40	0.10	5	0.71	2	5
11. Recognizes psychosocial aspects of illness	57	2	9	4.35	0.11	4.5	0.73	3	5
12. Manages patients with complex psychosocial problems*	59	0	31	4.21	0.13	4	0.69	3	5
13. Addresses comorbidities	58	1	8	4.40	0.10	4.5	0.67	3	5
14. Addresses non-pharmacological therapies (exercise, weight management, smoking cessation)	57	2	8	4.35	0.10	4	0.66	3	5
15. Shows compassion for patients and families	58	1	7	4.55	0.09	5	0.61	3	5
16. Respects the rights of patients	57	2	11	4.46	0.09	5	0.62	3	5
17. Manages healthcare resources efficiently	59	0	8	4.24	0.12	4	0.86	2	5

In addition to the video-based peer review and feedback, participating physicians deemed it important to review the physician’s consultation notes for context and to provide feedback on written communication. Cameras were placed a priori in each exam room to minimize intrusion and ensure patient privacy.

### Phase 2: medical colleague questionnaire ratings

Table 1 presents descriptive statistics outlining the results from the peer evaluation ratings assigned by each reviewer. Overall, physicians rated their colleagues highly. Of the 1003 ratings, there were only three cases when an individual was rated “below average”.

Reviewers were unable to respond to two of the 17 items included on the questionnaire, as these items focused on areas not covered in the patient visits. Specifically, these questions required the respondent to assess communication with patients’ families (item 2) and the physician’s ability to manage complex psychosocial problems (item 12).

The internal consistency of the ratings provided to physician performances were very high (*α* = 0.94), regardless of which round of ratings were considered (round 1 *α* = 0.89; round 2 *α* = 0.95; self-rating *α* = 0.95). Therefore, all subsequent analyses were done on the average score assigned to each physician to avoid problems of missing data. The average score across observed performances was 4.52 with a range from 3.60 to 5.00 (median = 4.70; S.D. = 0.47). Physicians rated their own performances slightly higher (mean = 4.70, 95% C.I. = 4.50–4.91) than did peer physician reviewers (mean = 4.43, 95% C.I. = 4.28–4.57); *F*_1,58_ = 4.81, *p* < .05.

**Fig. 2:**
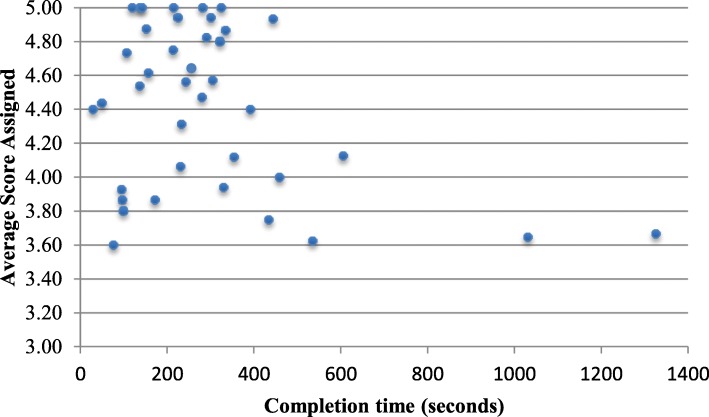
Average score assigned and response time

A negative correlation of *r* = − 0.36 was found between the amount of time taken to complete the rating task and the average score assigned (see Fig. 2). With the removal of two outliers (i.e., completion times that were greater than 1000 s), however, the correlation decreased to *r* = − 0.11. Physicians spent the same amount of time reviewing videos of themselves (mean = 274.5 s, SD = 357.42) relative to reviewing their colleagues’ performance (mean = 295.9 s, SD = 248.39); t (57) = 2.95, *p* = .769.

### Participant perceptions

As illustrated in Table 2, participants’ perceptions of the feedback they received were generally favourable.

**Table 2:** Participant Perceptions from the Peer Review Process

	Mean	Median	S.D.	Min	Max
1. Informative	4.0	4	0.71	3	5
2. Meaningful	3.6	4	1.14	2	5
3. Comprehensive	3.6	4	0.55	3	4
4. Credible	4.6	5	0.55	4	5
5. Anxiety provoking	2.8	2	1.10	2	4
6. Frustrating	2.4	2	0.89	2	4
7. Constructive	3.8	4	0.45	3	4
8. Useful	4.0	4	0.00	4	4
9. Communicated effectively	4.0	4	0.00	4	4
10. Idea generating	4.2	4	0.84	3	5
11. Likely to improve my practice	3.6	4	1.14	2	5
12. Motivating	4.0	4	0.71	3	5
13. Sufficient	3.6	4	0.55	3	4
14. Appropriate	3.8	4	1.10	2	5
15. Fair	4.0	4	0.71	3	5
16. Accurate	3.8	4	0.45	3	4
17. Valuable	4.2	4	0.84	3	5
18. Clear	4.0	4	0.00	4	4

### Phase 3: focus group

Phase 3 of the study investigated participants’ perceptions of the overall project, insights into how video-based peer feedback could improve their clinical practices and professional development, and recommendations. The themes that were identified through content analysis included: 1) equipment feasibility and software, 2) advantages of the peer-review video feedback process, and 3) disadvantages of the peer-review feedback process. The themes are described in further detail below with illustrative quotes.

### Equipment feasibility and software

The clinicians involved in this study believed video-based review to be less intrusive and more convenient than direct observation. One participant expressed: “I would find [an observing reviewer to be] intrusive … totally unnatural if there was somebody just sitting there and watching you”. While another participant added: “I don’t think patients would appreciate that either … having another person watching”. Incorporating the video recording into their office for the sake of submitting to a feedback process was quite feasible because the camera was small, inconspicuous, convenient, and easy to operate. Moreover, clinicians reported that patients were receptive to having the consultation recorded. Many favoured the video recording over an in-person reviewer because “it was not obtrusive to patients” and “you forget the camera is there”.

Regarding the software used for recording and review of annotated feedback, clinicians found the interface for reviewing peer videos to be helpful. Some of the features found to be particularly useful were the ability to fast forward through the video, to highlight specific instances of certain behaviours, and to incorporate annotations. However, some clinicians found the program to be challenging to use in the beginning and it took time to learn how to use the software properly as some of the features were not intuitive.

### Advantages of the peer-review video feedback process

One major benefit of the video-based feedback process frequently mentioned was the provision of accurate and detailed information regarding the patient-clinician interaction that a chart review alone would not be able to achieve (e.g., clinician’s communication skills). One person expressed the benefits of video review relative to chart review in this way:

“I think this is so much more valuable than a just a chart review. I think that the fact that it is in combination … the last consult note, the current consult note and the video, I think is much more information to learn from … even for one patient. Watch one patient interaction with two consult letters and an interaction are much more powerful than doing 10 charts.”

The recorded interactions allowed participants to gain insight into other communication methods and styles by seeing how their peers communicate with their patients and highlighting any specific areas from the consultation that were exemplary or areas that could be improved. In this way, they argued that the act of reviewing others might have been as (or even more) valuable to their own improvement than the opportunity to hear reviews about their own performance. Overall, participants found the video feedback to be complementary to chart reviews.

Through this process, many found that providing feedback was a good mentoring opportunity, and that this type of mentoring would improve their ability to provide better clinical care. One participant explained:

“Having to assess someone else is an important piece to this because I think that they gain something from looking at their own. We also gain something from actually observing somebody else and commenting on somebody else. I think that that process alone helps us improve because we all know around this table that teaching helps us improve our ability to provide clinical care.”

Participants had the ability to highlight and obtain consensus on specific behaviours. With annotations at exact points on the video, coupled with feedback from different reviewers of the same video, many respondents found that hearing the consensus of reviewers regarding certain behaviours was validating and reinforcing. From the reviewers’ perspective, participants indicated they found it beneficial to observe and comment on their colleagues’ performance.

Participants felt that, in general, there were few opportunities to view themselves in their daily practice. The ability to view their own performances via video recording was seen as a valuable opportunity to help them reflect. As one participant said:

“The main and most powerful element in this whole thing is getting to see yourself. You don’t know how you look like or how you act and you just keep doing the same thing you’ve been doing your whole life … And just being able to actually look at that video aside from all the comments and everything else, you just sort of pick up … oh, you know I can do that differently … that works well … That [video review] is super valuable and that every doctor should have it... it’s powerful”.

Another participant thought that observing his own performance allowed him to identify his own communication style:

“I actually think that evaluating myself is more valuable because … we all have a different style … the medical content I think everybody is fine, but the communication … everyone has their own style. And I think it’s nice to see how your style is because it’s different than what you think it is.”

### Disadvantages of the peer review video feedback process

Several drawbacks of the feedback process were brought up at the focus group that can be used to further improve the implementation of video-based feedback processes. Participants indicated that it might have been useful to have a follow-up with the reviewers, once all the feedback was reviewed, to discuss how to reconcile discrepancies between reviewers. Some participants believed the feedback received might have been more beneficial if they had not known each other (and been familiar with others’ practice patterns) prior to the study. Since the reviewers knew each other and had previous positive interactions, some individuals thought some of the comments might have been slightly biased and overly positive. As one participant expressed:

“I think that when I watch my own videos, independent of what people commented on, I would have commented on the same thing about myself that everybody else said about me. So, I actually don’t know how much extra value there was. Maybe we were just a group of people that were very nice and didn’t say what they really thought … It’s a small group, so … and you know each other and work together”.

Another disadvantage to the video recording was that patients may have been uncomfortable discussing negative psychosocial issues and thus may have avoided mentioning these issues during the recorded encounter. Lastly, the time required to review the peer videos and provide feedback may have been too long due to having too many videos to review, and annotating various relevant sections being rather time-intensive.

## Discussion

We aimed to explore the application of video-based feedback as a way to support continuing professional development and performance improvement among peer clinicians. Using a participatory action research approach we sought to understand how actively practicing physicians might consider using video-based feedback effectively, pre-experience, and what issues they perceived, post-experience, to guide the iterative improvement of this intervention. Overall, our results illustrated that peer clinicians reviewing and annotating video performances using a multi-source feedback questionnaire was a feasible and acceptable approach to support professional development. The study was able to outline advantages of video-based feedback as a convenient way to provide comments to others, observe accurate and detailed information, and self-reflect on one’s own practices. At the same time it highlighted the importance of remaining conscious of time requirements for the review process [8].

Participants believed the process provided an opportunity to learn from patient consultations by allowing peers to mentor one another. Their perspectives suggested that the ability to provide and receive feedback on specific behaviours and obtain an overall consensus from the group had a meaningful impact on their practice. Despite the advantages of having peer reviewers with previously established rapport, the participants suggested that having peers who were familiar with each other would not be objective enough as an approach to peer assessment in other contexts. Therefore, we would recommend that the review process incorporate a mixture of reviewers with varying levels of familiarity. That said, peer review in the form we conducted may not be suited to a formal evaluation process such as a high stakes revalidation process conducted by a licensing body and, as such, we consider the benefits of inducing feedback and reflection to be more central to the benefits of video-based review than the objective assignment of ratings.

Our findings also highlight the importance of designing a process with which physicians will engage as the process of reviewing videos was commented upon as time intensive for participants and the software for annotation found to be challenging for some. Disengagement of participants due to technical difficulties might compromise the quality of feedback produced. Our study did not specifically examine this issue, but one would surmise that using this video method would be less time intensive than if reviewers were required to be physically present to observe the interactions between those being reviewed and their patients.

### Limitations

Several limitations of our study design should be taken into account. First, the participating rheumatologists were selected purposefully as those who could serve as key informants given their educational interests and activity. As such, they are not likely to be representative of all physicians, which will limit the generalizability of our findings. This trade-off was considered worth making, given the purpose of the study, as it allowed for robust participatory inquiry, self-reflection and critique from an informed pedagogical perspective.

Similarly, the patient interactions reviewed for this study were limited to follow-up visits rather than new patient encounters. The physicians already having background knowledge of the particular patients enrolled may have contributed to the relatively high ratings observed and, in turn, to the positive feelings regarding the value of the feedback processes. Video-based feedback for new visits would be different in that the physician would be assessed for a variety of other competencies including basic history-taking skills, establishing rapport, information gathering and initial discussion of the differential diagnosis and plan. Whether or not such benefits are greater than the potential disadvantages that led our physician participants to want to focus on follow-up visits remains to be seen.

Third, our study was not designed to offer a definitive answer as to whether or not planned changes were implemented. Participants claimed the opportunity to review their own videos offered a helpful trigger for self-reflection and motivated intention to change. That, coupled with congruous advice from reviewers, could form the powerful alliance required to cause actual change in clinical behaviour, but we cannot say that took place with certainty.

Fourth, the asynchronous nature of our pilot is also worth commenting upon, as participants expressed interest in having follow-up discussions with reviewers to go over certain suggestions. While our study did not examine this possibility, adding an interactive component where the reviewers and those being reviewed can interact after the video reviews, in person, via telephone, or by desktop videoconferencing, may further enrich the experience for those being reviewed.

## Conclusions

Overall, video-based peer consultations offer a feasible and acceptable way to support professional development and quality improvement among a group of clinical peers. The findings further suggest video recordings of patient encounters to be useful tools to help physicians self-reflect on their own clinical practice. This particular approach was able to illustrate the benefit of providing a non-intrusive method for clinicians to view their own patient consultations while gaining feedback from peers, which ultimately acts as a valuable tool for clinical practice improvement. Allowing clinicians to reflect asynchronously on their performance reduces time and logistical constraints that may lessen the capacity to engage in direct observation of peers, but participants still saw value in creating opportunity to obtain clarification regarding the feedback received. At a minimum, video-based feedback creates a stimulus for reflection that might enable physicians themselves to follow up on issues about which they would value more dialogue. Further evaluating this model beyond pilot testing for its efficacy to promote self-reflection and openness to coaching would be worthwhile.

## Data Availability

The datasets generated and/or analysed during the current study are not publicly available due to the informed consent outlined in the approved ethics review, but are available from the corresponding author on reasonable request.
